# Supplementary figures and images for: Automatic recognition of complementary strands: lessons regarding machine learning abilities in RNA folding
Source: Front Genet. 2023 Sep 4;14:1254226. doi: 10.3389/fgene.2023.1254226 (PMC10507318; doi:10.3389/fgene.2023.1254226)

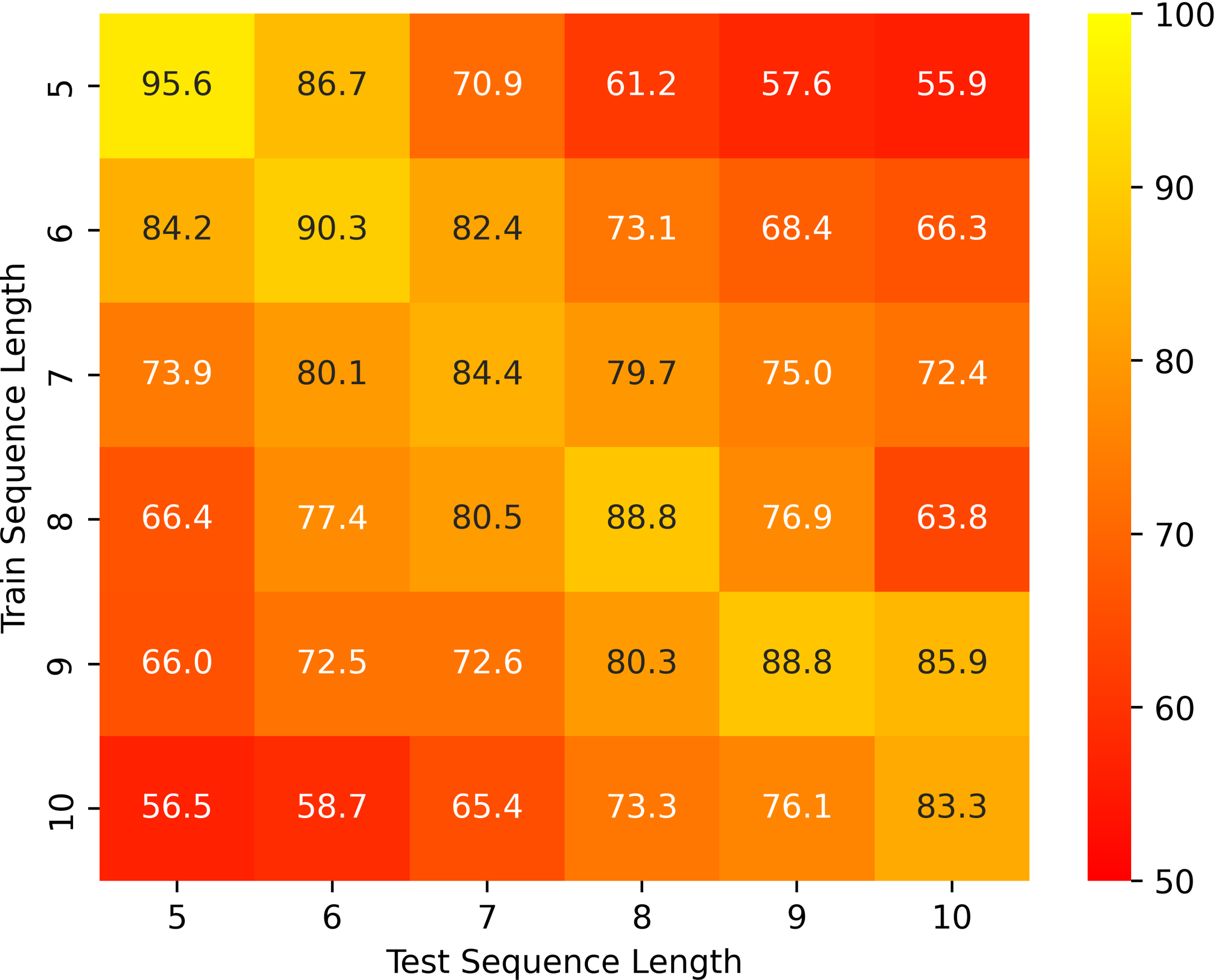

Supplement: Supplementary file 1 [file Image4.png]

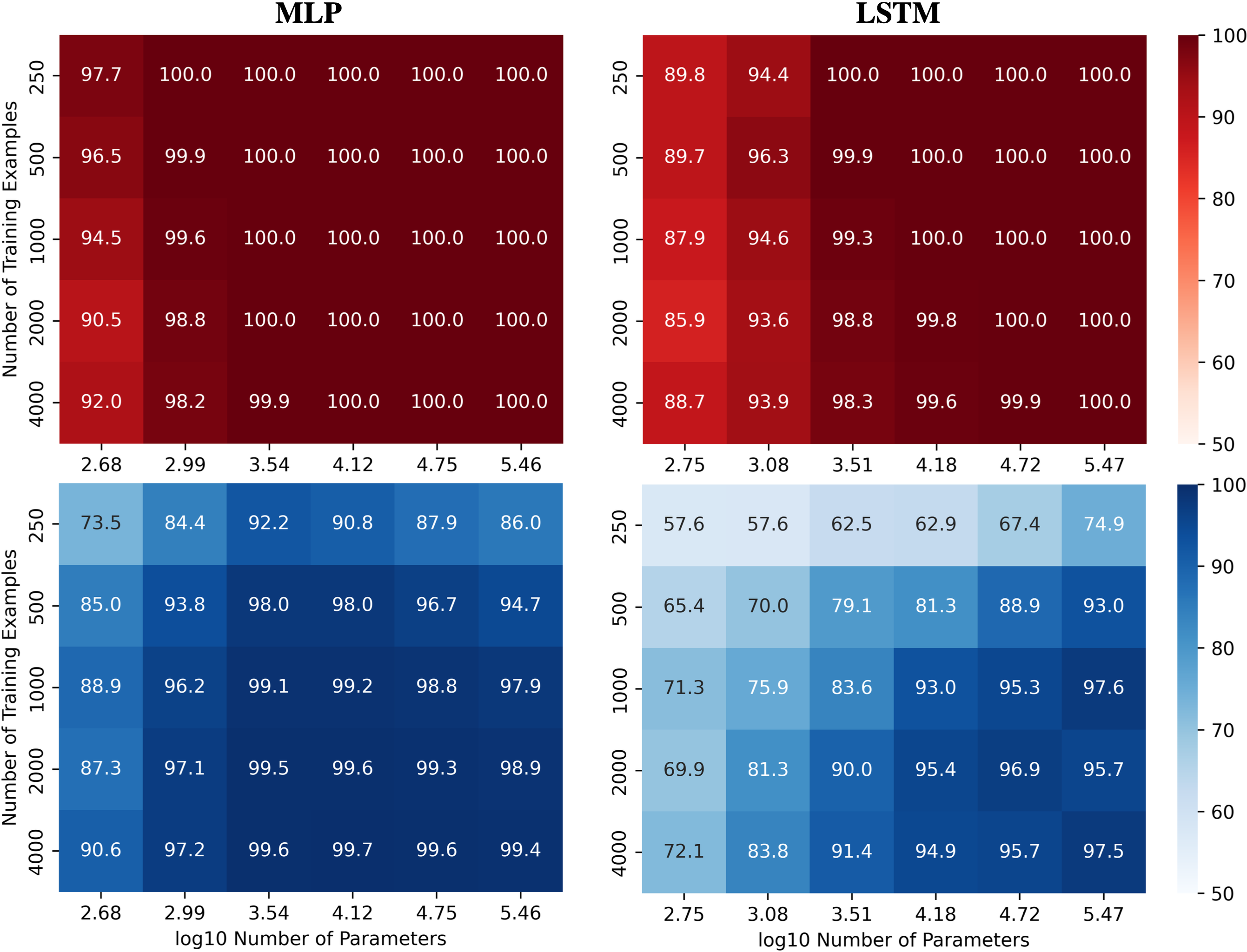

Supplement: Supplementary file 2 [file Image2.png]

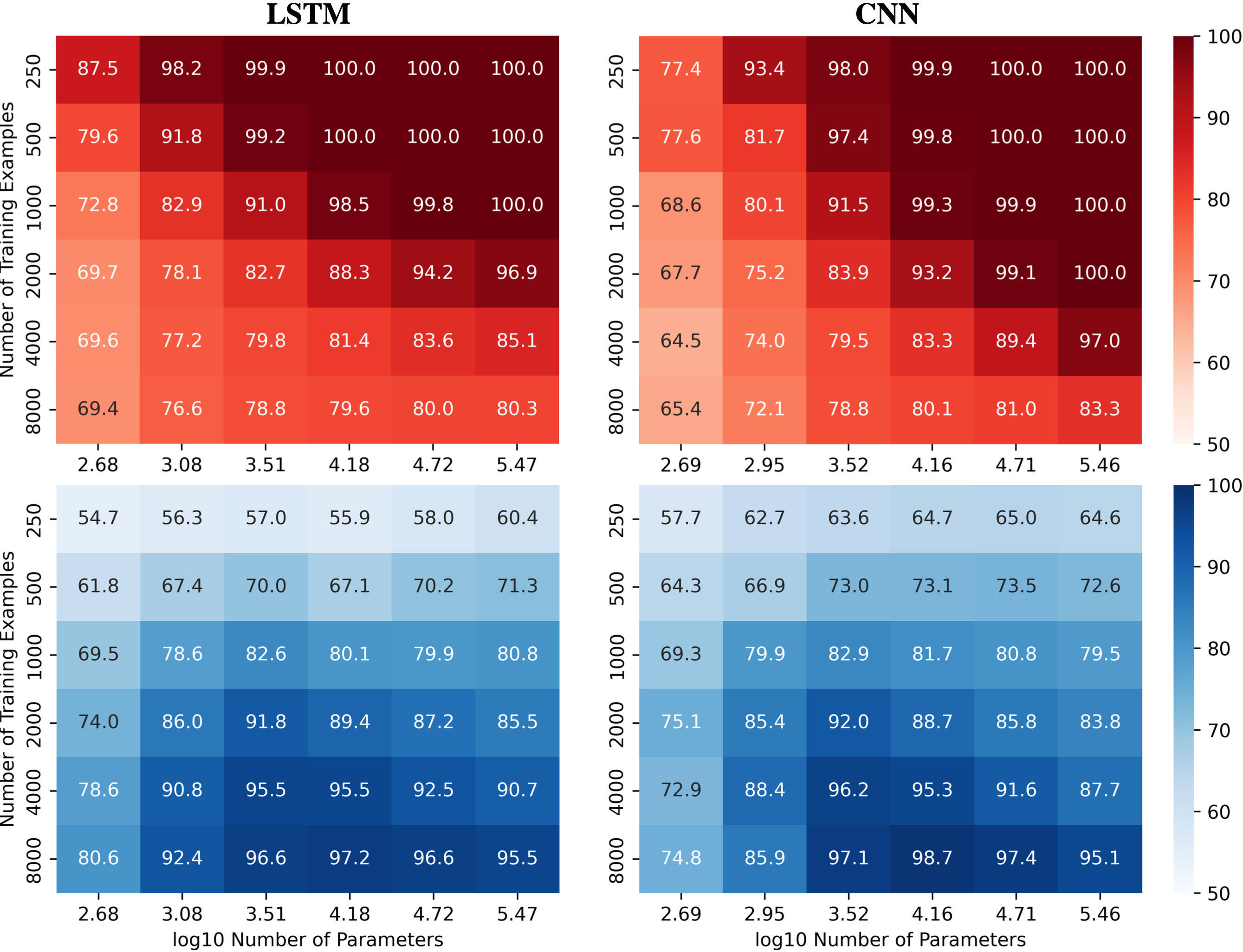

Supplement: Supplementary file 3 [file Image1.png]

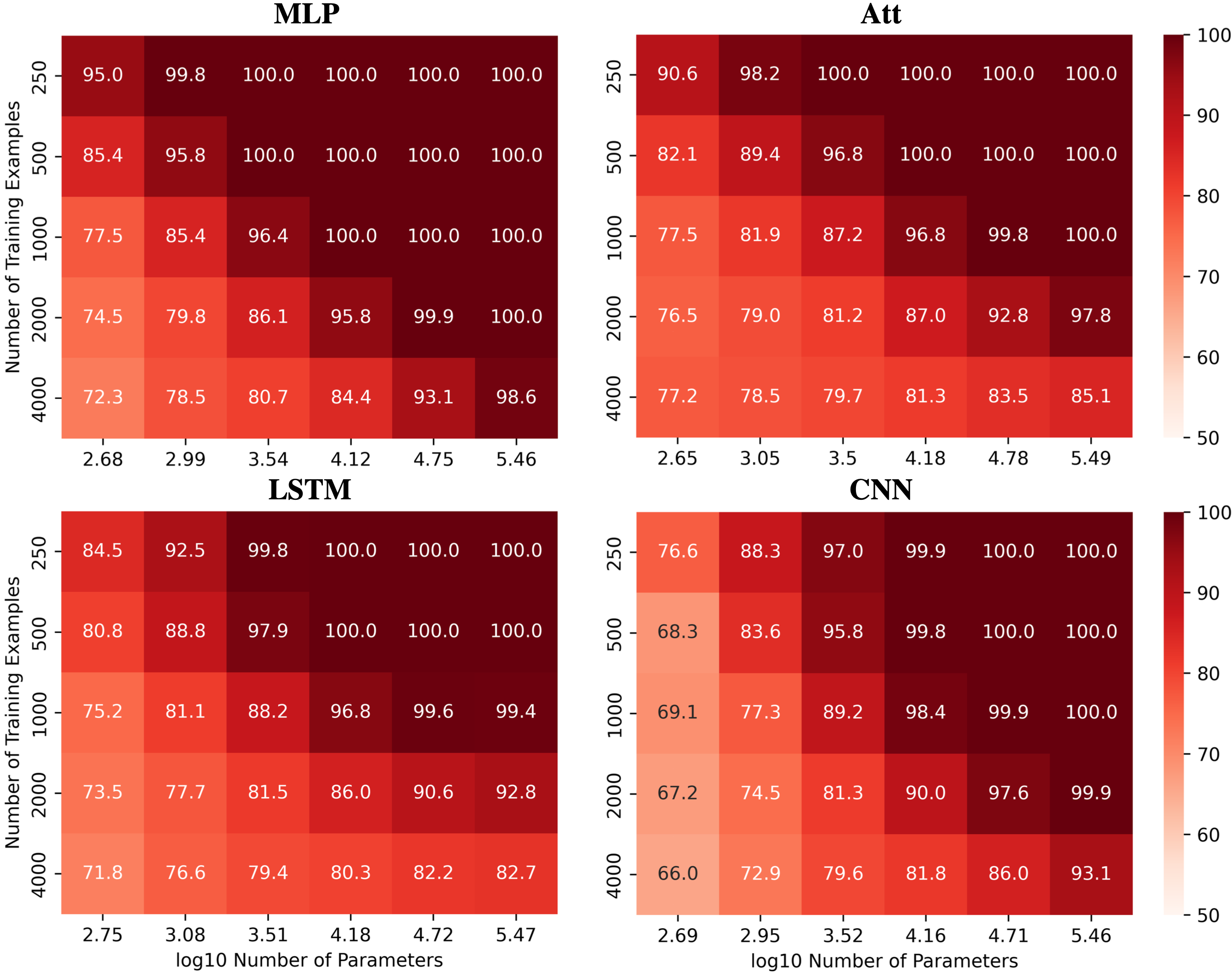

Supplement: Supplementary file 4 [file Image3.png]
